# Predicting skin permeability using HuskinDB

**DOI:** 10.1038/s41597-022-01698-4

**Published:** 2022-09-23

**Authors:** Laura J. Waters, Xin Ling Quah

**Affiliations:** School of Applied Sciences, University of Huddersfield, Queensgate, Huddersfield, HD1 3DH UK

**Keywords:** Biophysical chemistry, Bioanalytical chemistry

## Abstract

A freely accessible database has recently been released that provides measurements available in the literature on human skin permeation data, known as the ‘Human Skin Database – HuskinDB’. Although this database is extremely useful for sourcing permeation data to help with toxicity and efficacy determination, it cannot be beneficial when wishing to consider unlisted, or novel compounds. This study undertakes analysis of the data from within HuskinDB to create a model that predicts permeation for any compound (within the range of properties used to create the model). Using permeability coefficient (*K*_p_) data from within this resource, several models were established for *K*_p_ values for compounds of interest by varying the experimental parameters chosen and using standard physicochemical data. Multiple regression analysis facilitated creation of one particularly successful model to predict *K*_p_ through human skin based only on three chemical properties. The model transforms the dataset from simply a resource of information to a more beneficial model that can be used to replace permeation testing for a wide range of compounds.

## Introduction

Permeation of a compound through human skin is an increasingly important delivery route in pharmaceutical applications as well as being a vital property to consider in risk assessment analysis for any compound that may come in to contact with skin. *In vivo* analysis can be a good predictor of properties, such as bioavailability^1,2^, where human volunteers are used for stratum corneum (SC) sampling but often *in vitro* techniques are used instead. It is apparent that discrepancies often occur between the *in vivo* reality and *in vitro* based predicted data. One reason for this is the variety of experimental data, in terms of skin origin or age and experimental conditions. Even when SC sampling is used the situation can be complex^3^. In the vast majority of such published studies experimental data facilitates calculation of the permeability coefficient (*K*_p_) whereby a compound permeates through skin under steady-state conditions from an aqueous vehicle. To minimise potential variability in data from the source of skin, alternatives have been investigated including work in our group using poly(dimethylsiloxane) as a skin replacement^4–6^ and alternative analytical techniques including micellar liquid chromatography^7^. However, all of these experimental systems require extensive laboratory work to allow measurement of *K*_p_. To avoid this time-consuming process, predictive models are frequently used for modelling in drug development, such as for pharmacokinetic applications^8^ and of relevance to this work, for skin permeability. These models utilise the calculated *K*_p_ value to formulate a quantitative structure-permeability relationship (QSPR) that relates permeability to identified physicochemical properties of the compound undergoing permeation, such as hydrogen bond activity^9^. With over thirty years of research in this area there are numerous datasets available for skin absorption modelling studies, ranging in many parameters including the number of compounds considered, the source and thickness of skin used, experimental temperature, pH and vehicle composition^10–13^. Prior to this study, the vast majority of data used in creating permeation models is based on animal skin studies which are renowned for being poor mimics for human skin, a phenomenon known as the inter-species translational gap^14,15^. One study in particular found that only four of the thirty three QSPRs available at the time of publication were deemed ‘acceptable’ according to their stated four criteria with only one of these providing ‘reasonable’ predictions^16^. Since then, many QSPRs have been created varying in complexity with a particularly well-known model published in 1992 by Potts and Guy^17^ using a compounds partition coefficient (logP) and molecular weight (MW) to create the following (relatively simple) Eq. (1):

1 $$\begin{array}{l}{\rm{\log }}{K}_{{\rm{p}}}=0.71\;{\rm{logP}}-0.0061\;{\rm{MW}}-6.3\\ {\rm{n}}=93,\;{r}^{2}=0.67\end{array}$$

Other, often more elaborate models have incorporated additional physicochemical properties to predict *K*_p_ including hydrogen bond acceptor/donor activity, solubility, charge, melting point, polarisability and vehicle formulation^18^. However, the variability in experimental conditions undertaken to create these datasets has been noted, such as if the solute is analysed under finite or infinite dosing conditions^19^ and the chosen experimental volume^20^. These effects can result in variability in dataset *K*_p_ values which will ultimately affect the suitability and success of the model created^21–23^. It has therefore been stated that ideally values should be obtained under the same experimental conditions^24^ yet this is difficult to achieve in reality. Over the years many datasets have been employed to predict percutaneous absorption and in some cases combined to create even more detailed datasets^25^.

In 2020, a new dataset was created (HuskinDB^26^) that removed the uncertainty associated with previous models which was revolutionary in its approach^27^ and, since a recent update, has been expanded even further. The freely accessible database now lists skin permeation values (*K*_p_ values) for 253 compounds analysed with human skin yet it also includes experimental parameters such as skin source site, skin layer used, preparation technique, storage conditions along with experimental conditions such as temperature of the acceptor and donor solutions, pH and solution compositions (where available) for each *K*_p_ value quoted. Since its release this database has been used by regulatory agencies for dermal risk assessment^28^ and is becoming known as a useful resource to researchers^29^.

This study utilises this in-depth dataset to create unique models that take in to account this additional information, highlighting the influence of experimental parameters on data analysis and leading to a highly specific and optimised model for use when investigating new compounds for permeability.

## Results and Discussion

With 550 *K*_p_ values in total, HuskinDB is a significant source of data for those wishing to know the extent of skin permeation for any of the 253 compounds included. It has many benefits to those using the dataset, firstly that all included data was obtained using human skin thus variability is limited compared with other datasets that have included animal and other non-human membranes in the analysis. Furthermore, a variety of experimental parameters are included for each *K*_p_ value allowing the researcher to obtain a specific *K*_p_ value under whatever specific conditions are of interest, such as temperature and donor concentration. However, this work takes the dataset much further and utilises the data to create models that then permit *prediction* of *K*_p_ for other compounds of interest rather than limited to only those in the dataset. This is particularly beneficial for several applications, including when considering compounds that currently exist (but are not already in the dataset) or, have not yet even been synthesised.

The 27 scenarios where data was available from the dataset were each analysed to create a QSPR model and are listed in Table 1.

**Table 1:** QSPR models for skin permeability (*K*_p_) coefficient prediction using data extracted from HuskinDB, as related to a compound’s partition coefficient (logP), topological surface area (TPSA) and molecular volume (MV).

Skin Souce	Skin Type	Donor Conc.	Exp. Temp (°C)	No. of cmpds	R^2^	Equation
Breast	Epidermis	Diluted	36–40	9	0.9839	LogKp = −2.869 - 0.406 LogP - 0.143 TPSA + 0.021 MV
Breast	Epidermis + Dermis	Saturated	36–40	6	0.8321	LogKp = −3.083 - 0.728 LogP - 0.168 TPSA + 0.043 MV
Breast	Epidermis + Dermis	Diluted	20–25	4	0.9703	LogKp = −4.993 - 0.808 LogP - 0.168 TPSA + 0.033 MV
Breast	Epidermis + Dermis	Diluted	31–35	20	0.5464	LogKp = −4.406 - 0.514 LogP - 0.004 TPSA - 0.006 MV
Breast	Epidermis + Dermis	Diluted	36–40	5	0.8747	LogKp = −6.297 - 3.151 LogP - 0.424 TPSA + 0.128 MV
Abdomen	Epidermis	Saturated	20–25	10	0.9595	LogKp = −5.203 + 7.125 LogP + 0.525 TPSA - 0.236 MV
Abdomen	Epidermis	Saturated	26–30	8	0.9772	LogKp = −6.875 + 1.410 LogP + 0.206 TPSA - 0.059 MV
Abdomen	Epidermis	Diluted	20–25	36	0.8545	LogKp = −6.052 + 0.777 LogP - 0.004 TPSA - 0.008 MV
Abdomen	Epidermis	Diluted	26–30	2	N/A	LogKp = −6.477 + 1.467 LogP + 0.220 TPSA - 0.051 MV
Abdomen	Epidermis	Diluted	31–35	36	0.7102	LogKp = −5.788 + 0.099 LogP - 0.028 TPSA + 0.008 MV
Abdomen	Epidermis	Diluted	36–40	43	0.1619	LogKp = −6.231 + 0.211 LogP - 0.006 TPSA - 0.001 MV
Abdomen	Dermis	Saturated	20–25	8	0.9737	LogKp = −6.277 + 1.778 LogP + 0.226 TPSA - 0.077 MV
Abdomen	Dermis	Diluted	20–25	16	0.8334	LogKp = −4.584 + 0.931 LogP + 0.019 TPSA - 0.020 MV
Abdomen	Dermis	Diluted	31–35	6	0.9393	LogKp = −1.784 + 2.499 LogP + 0.040 TPSA - 0.081 MV
Abdomen	Epidermis + Dermis	Saturated	26–30	4	0.5268	LogKp = −7.599 - 1.743 LogP - 0.113 TPSA + 0.045 MV
Abdomen	Epidermis + Dermis	Diluted	20–25	4	0.9916	LogKp = −2.586 + 2.453 LogP - 0.082 TPSA - 0.053 MV
Abdomen	Epidermis + Dermis	Diluted	26–30	8	0.8166	LogKp = −5.361 + 0.400 LogP - 0.025 TPSA - 0.001 MV
Abdomen	Epidermis + Dermis	Diluted	31–35	45	0.1422	LogKp = −6.129 + 0.193 LogP + 0.004 TPSA - 0.006 MV
Abdomen	Epidermis + Dermis	Diluted	36–40	14	0.9734	LogKp = −6.960 + 0.975 LogP - 0.004 TPSA - 0.005 MV
Abdomen	Stratum corneum	Diluted	26–30	3	1	LogKp = −5.695 - 1.147 LogP - 0.137 TPSA + 0.047 MV
Abdomen	Stratum corneum	Diluted	31–35	3	1	LogKp = −6.009 - 0.043 LogP - 0.036 TPSA + 0.008 MV
Thigh	Epidermis	Diluted	31–35	3	1	LogKp = −13.211 - 1.366 LogP + 0.031 TPSA + 0.031 MV
Thigh	Epidermis	Diluted	36–40	3	1	LogKp = −11.084 - 1.472 LogP - 0.144 TPSA + 0.043 MV
Thigh	Epidermis + Dermis	Diluted	20–25	5	0.9344	LogKp = 2.734 + 3.503 LogP + 0.007 TPSA - 0.088 MV
Thigh	Epidermis + Dermis	Diluted	26–30	17	0.6139	LogKp = −6.826 + 0.656 LogP + 0.006 TPSA - 0.006 MV
Thigh	Epidermis + Dermis	Diluted	31–35	3	1	LogKp = −4.001 + 0.309 LogP - 0.085 TPSA + 0.031 MV
Thigh	Epidermis + Dermis	Diluted	36–40	21	0.3329	LogKp = −6.876 + 0.566 LogP - 0.002 TPSA - 0.002 MV

With respect to skin source, i.e. anatomical site, it is known that the source can affect permeation^30^. Only five scenarios were analysed using breast skin and six with thigh skin, leaving the majority (sixteen) using abdomen skin. This is as expected as skin from the abdomen is frequently used in analysis for convenience reasons^31^. For permeation analysis, skin can be separated into layers to allow researchers to focus on permeation through only the epidermis or dermis, both epidermis and dermis combined or the stratum corneum. Nine of the scenarios analysed the epidermis only, three the dermis only, thirteen the epidermis and dermis and two the stratum corneum. It could be argued that permeation through the stratum corneum is the most important layer to consider as this is the first stage of the process and will therefore dictate subsequent permeation. However, as permeation must also be achieved through the entire epidermis and then dermis it is also arguable that analysis should consider both layers combined, as was the case for the majority of the scenarios. With respect to donor concentration, twenty two of the scenarios involved a diluted solute concentration in the donor phase with the remaining five as neat (saturated) solutions. This finding is particularly interesting as it is more usual in permeation analysis to apply saturated solutions to the skin to maintain sink conditions throughout the experiment^32^. Finally, experimental donor solution temperature was particularly variable throughout the dataset thus a decision was made to divide the experimental data into four options to simplify analysis. Results appeared equally split in that seven scenarios involved an experimental donor solution temperature between 20 and 25 °C, six between 26 and 30 °C, seven between 31 and 35 °C, with seven between 36 and 40 °C. This finding was surprising if the data entered in the dataset was acquired for *in vivo* prediction as the surface of skin is usually approximately 33 °C, and internal body temperature 37 °C^33,34^. Therefore, the *in vivo* permeation process will occur between these temperatures and the latter two temperature options of the four listed would be the most suitable choices rather than the two lower temperature options.

Originally, 96 scenarios were considered using the four variables discussed yet a lack of data (where no compounds fit the criteria) for 69 scenarios reduced the number of models created to 27. Of these 27 remaining scenarios, 19 had a limited number of compounds (n = ≤15) which was deemed too low for consideration as a suitable QSPR model. The eight remaining scenarios therefore contained 16 or more compounds with a maximum number of 45 compounds.

Along with ensuring a suitable number of compounds had *K*_p_ values available to create the QSPR model, the coefficient of determination (R^2^) was an important factor for consideration with a value approaching 1 sought. This concept, whereby the value is as close to 1 as possible, has often been the focus of discussions surrounding the suitability of models for permeability prediction. Although absolute limits on what can be classed as an ‘acceptable threshold’ do not exist, researchers have previously described values below 0.3 as poor^16^, around 0.6 as significant^32^ and above 0.8 as good^25^. Values in this study for R^2^ ranged from 0.1422 (i.e. very little correlation) up to 0.8545 (i.e. an acceptable correlation). An ideal model would combine the greatest number of compounds possible with the highest R^2^ value yet in reality this is not always possible. As a consequence, a compromise between these two factors was applied and the most suitable model from those available deemed to be that which included 36 compounds with an R^2^ value of 0.8545. For further confirmation of the performance of this model, the total dataset (n = 36) was subdivided into two groups: a training set (n = 29) and a test set (n = 7) with the latter chosen at random then checked to ensure it included a range of logP, TPSA and MV values. Equation (2) displays the equation created as a result of this process with the training and test set coefficients of determination (R^2^) and root mean square error values (RMSE) specified.

2 $$\begin{array}{l}{\rm{\log }}{K}_{{\rm{p}}}=-6.136+0.818\;\log {\rm{P}}-0.005\;{\rm{TPSA}}-0.007\;{\rm{MV}}\\ {\rm{Training}}\;{\rm{set}}:{\rm{n}}=29{\rm{,}}\;{{\rm{R}}}^{2}=0.8428,\;{\rm{RMSE}}=0.30\\ {\rm{Test}}\;{\rm{set}}:{\rm{n}}=7,{{\rm{R}}}^{2}=0.8949,\;{\rm{RMSE}}=0.35\end{array}$$

Interestingly, this particular scenario was not for full thickness skin but epidermis only, with a diluted donor phase and at the lowest of the four donor solution temperature ranges considered. Why this particular model achieved the best performance of all the models created is unclear at this time. However, the high level of control over skin choice, anatomical site, skin thickness, donor phase concentration and experimental temperature do prove that removing variability in data can lead to a model with high predictive ability.

Although the derived R^2^ value is deemed adequate, it could be argued that the comparatively small dataset utilised may reduce the acceptability of the model for permeation prediction in a more general context. To consider an alternative approach (whereby a larger dataset was used) an additional QSPR model was created to investigate how this compares with Eq. (2). In this additional model any compound with a *K*_p_ value was included although if multiple values were available for a compound, four experimental variables were used to reduce the number to one. These were set as: abdomen site, epidermis and dermis layers, concentrated solute, experimental donor solution temperature 30–35 °C, as well as an experimental pH between 7 and 7.5. Using these criteria all 253 compounds were analysed and found to have a low coefficient of determination where R^2^ = 0.2308. This could be improved somewhat by removing any predicted log*K*_p_ values that were more than ± 1.5 from the dataset value, i.e. the extreme outliers, to produce a more acceptable model (Eq. (3)) with 214 compounds included and a coefficient of determination of R^2^ = 0.5044. The vast majority of the 39 compounds that were deemed ‘outliers’, and therefore removed to create Eq. (3), were at the extremities of the *K*_p_ values considered. As before, the total dataset (n = 214) was subdivided into two groups: a training set (n = 171) and a test set (n = 43) with the latter chosen at random and then checked to ensure it included a range of logP, TPSA and MV values. Equation (3) displays the equation created as a result of this process with the training and test set coefficients of determination (R^2^) and root mean square error values (RMSE) specified.

3 $$\begin{array}{l}{\rm{\log }}{K}_{{\rm{p}}}=-5.820+0.319\;\log {\rm{P}}-0.001\;{\rm{TPSA}}-0.005\;{\rm{MV}}\\ {\rm{Training}}\;{\rm{set}}:{\rm{n}}=171{\rm{,}}\;{{\rm{R}}}^{2}=0.5042,\;{\rm{RMSE}}=0.73\\ {\rm{Test}}\;{\rm{set}}:{\rm{n}}=43,\;{{\rm{R}}}^{2}=0.5057,\;{\rm{RMSE}}=0.84\end{array}$$

**Fig. 1:**
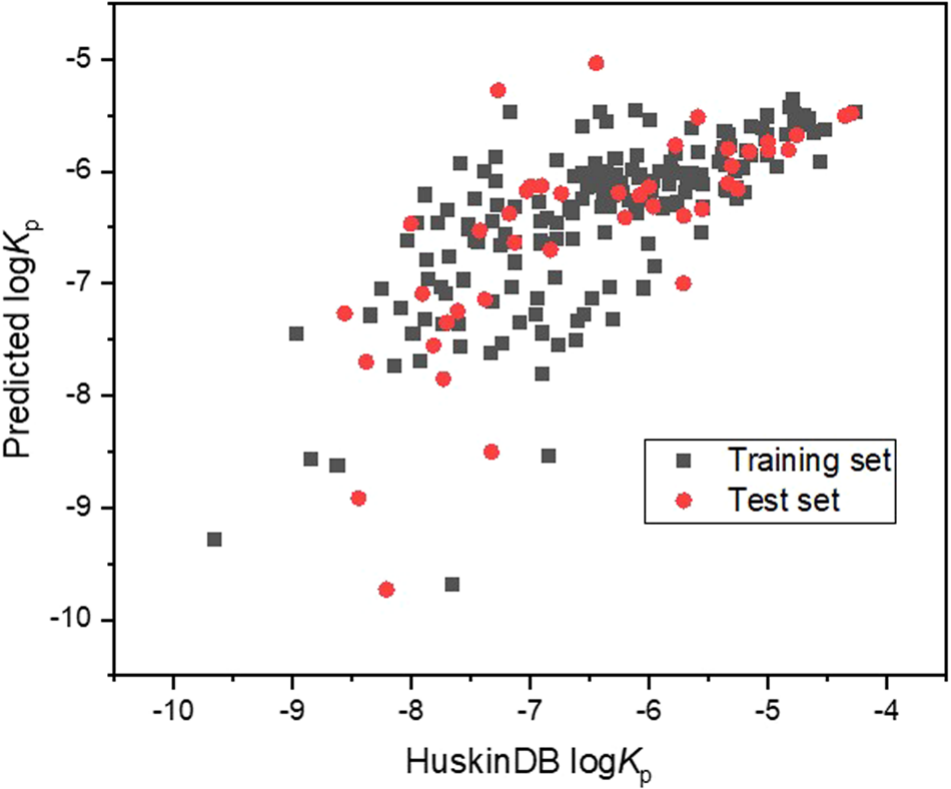
Predicted (from Eq. (3)) and experimental (HuskinDB) log*K*_p_ values for the training and test sets.

Figure (1) displays the relationship between the predicted and experimental log*K*_p_ values for the 214 compounds analysed using Eq. (3) based upon HuskinDB logarithmic *K*p values expressed in cm/s.

Although Eq. (3) is superior in that a far larger dataset was included, the lower coefficient of determination (and higher RMSE) indicates that it would be better to use Eq. (2) rather than Eq. (3) when attempting to predict permeability coefficients.

Many models already exist for predicting skin permeation, including the DERMWIN™ model (https://www.epa.gov/tsca-screening-tools/epi-suitetm-estimation-program-interface). This model is based on an equation similar to that proposed by Potts and Guy^17^ in that it uses the same physicochemical properties to predict log*K*_p_ values and is often used by researchers for comparison with newly proposed models^21,35,36^. For comparative analysis in this work, log*K*_p_ values for the 214 compounds were analysed using DERMWIN™ and the values obtained compared with those from HuskinDB (as selected for Eq. 3). It was found that the coefficient of determination between these two sets of log*K*_p_ values was lower than both the training and test set values presented in Eq. 3 (0.4351 for DERMWIN™ vs. HuskinDB and 0.5042 then 0.5057 for Eq. 3 vs. HuskinDB), along with a higher RMSE (1.04 for the former and 0.73 then 0.84 for the latter). It can therefore be concluded that Eq. 3 provides a superior model when predicting log*K*_p_ values for human skin permeation compared with the DERMWIN™ model.

In summary, HuskinDB is an exciting and useful new database providing permeability data for a large range of compounds. This extensive dataset can be of even more use by creating models using the plethora of experimental information available about each *K*_p_ value. It would appear that the most successful QSPR model utilised a total of 36 compounds with four specified experimental conditions to create an *in silico* method for predicting permeation for any compound of interest. In comparison, a larger dataset can be considered with less focus on experimental variable selection to create an alternative model yet with a lower degree of correlation achievable. In both cases, this expansion of HuskinDB to allow prediction of permeation for compounds not included in the dataset is an exciting development in permeation prediction. This takes the database from being a limited resource only for included compounds to a way of predicting permeation for any compound of interest. As further experimental data becomes available in literature over the following years (with the required experimental parameters listed) then it will be possible to expand the dataset even further, thus potentially creating an even more successful model for prediction of permeation than that proposed in this study.

## Methods

All *K*_p_ values included in this study were extracted from HuskinDB^27^, expressed as logarithmic *K*p values measured in cm/s. Four experimental variables were selected that were deemed particularly influential on the *K*_p_ value obtained (and encompassed a comparatively large total quantity of compounds), namely: skin source (breast/abdomen/thigh), skin layer used (epidermis/dermis/epidermis + dermis/stratum corneum), concentration of donor solution (neat/diluted) and donor solution temperature (divided in to 20–25/26–30/31–35/36–40 °C). This created a total of 96 experimental scenarios with associated *K*_p_ values encompassing all possible combinations (3 for skin source × 4 for skin layer × 2 for concentration and × 4 for temperature). However, of the 253 compounds in the dataset, 71 were excluded as they did not have specified experimental conditions for at least one of the four variables under investigation. Furthermore, of the 96 scenarios only 27 included one or more *K*_p_ values (i.e. n ≠ 0) reducing the dataset further. When more than one *K*_p_ value remained (even after applying the four variables) then all values were included in the analysis. These *K*_p_ values were then analysed against three physicochemical properties for each compound: partition coefficient (logP), topological polar surface area (TPSA) and molecular volume (MV) as these are known to be influential properties when determining *K*_p_ values^37^, particularly logP and MV as discussed by Tsakovska *et al*.^11^. Data for these three properties was extracted from Molinspiration (www.molinspiration.com, Molinspiration Cheminformatics, 2022). Multiple linear regression analysis (using Excel Solver) for each scenario created a series of unique QSPR equations with associated coefficients of determination (R^2^). Two equations in particular were then analysed in more detail whereby the data was divided into a training and test set for further validation of their performance. In both cases the total number of *K*_p_ values were randomly divided into two groups with ~ 80% in the training set and the remaining ~ 20% as a test set, ensuring a range of logP, TPSA and MV values were included in all cases.

## Data Availability

The datasets analysed during the current study are freely accessible from https://huskindb.drug350 design.de or 10.7303/syn21998881^26^.

## Code availability

No custom code was used to generate or process the data described in this manuscript.
